# TOGETHER, WE DO SAVE LIVES: EVALUATING THE IMPACT OF A GERIATRIC TRAUMA PROGRAM

**DOI:** 10.1093/geroni/igae098.4095

**Published:** 2024-12-31

**Authors:** Sandra Sanchez-Reilly, Erin Sidle, Shannon Crenshaw, Elizabeth Reilly, Marla Khalikov, Jennifer LaCoss, Nicolina Martinez, Ashley McGinity

**Affiliations:** South Texas Veterans Health Care System and the University of Texas Health Science Center at San Antonio, San Antonio, Texas, United States; University Health, San Antonio, Texas, United States; University Health, San Antonio, Texas, United States; Health Careers High School, Health Careers High School, Texas, United States; Health Careers High School, Health Careers High School, Texas, United States; Health Careers High School, Health Careers High School, Texas, United States; Health Careers High School, Health Careers High School, Texas, United States; UT Health San Antonio, UT Health San Antonio, Texas, United States

## Abstract

**Background:**

Trauma, the fifth cause of death among Older Adults (OA), accounts for up to 25% of all trauma admissions. Multi-comorbidities, polypharmacy, decreased function, and increased mortality are some challenges requiring geriatric expertise. Objective: To evaluate the clinical effectiveness of a geriatric trauma service consultation (GTSC) program in a level-1-trauma hospital.

**Methods:**

1321 GTSC were collected December/22-June/24. Variables included length-of-stay, morbidity/mortality and place-of-discharge.

**Results:**

Median age 78 (73-85). 58% female (765); 73% White, 20% Hispanic. 62% GTSC occurred within the first day of hospital admission, but 2% GTSC occurred >6 days after admission (delayed consults-DC). Within 30-days-of-admission, 3% and 2% hospital-readmission-rate. Falls constituted 74% GTSC, 17% were motor vehicle accidents. 87% GTSC came from home but only 35% returned home with 56% discharged to rehabilitation facilities. When comparing GTSC performed within 2 days of admission vs. DC, DC showed higher mortality (3.1% vs. 6.9%, p=0.24,NS); higher ICU stays (38.8% vs. 75%,p< 0.0001); higher 30-day-hospital-readmission (2.1% vs. 3.4%,p=0.47,NS); higher total length-of-stay (mean 6.9 days vs. 24.5,p< 0.0001); higher length-of-stay from day-of-GTSC (mean 6 days vs. 10.5,p=0.0004). OA with delirium had the longest hospital length-of-stay (mean 21.3 days,p< 0.0001) and higher ICU length-of-stay (15.9 days,p< 0.0001). Conclusions: When consulted early, GTSC showed to decrease mortality, hospital readmissions; and being significantly effective in decreasing ICU time and lowering length-of-stay among OA. Delirium poses a serious dilemma showing significantly worse length-of-stay and ICU need. Further studies need to focus on GTSC as an earlier intervention and to target specific geriatric syndromes such as delirium.

